# Evolutionary lability underlies drought adaptation of Australian shrubs along aridity gradients

**DOI:** 10.3389/fpls.2022.949531

**Published:** 2022-10-07

**Authors:** Gui-Qing Xu, Gaurav S. Kandlikar, Marcel C. Vaz

**Affiliations:** ^1^ State Key Laboratory of Desert and Oasis Ecology, Xinjiang Institute of Ecology and Geography, Chinese Academy of Sciences, Urumqi, China; ^2^ Division of Biological Sciences and Division of Plant Sciences, University of Missouri, Columbia, MO, United States; ^3^ Wilkes University, Institute for Environmental Science and Sustainability, Wilkes-Barre, PA, United States

**Keywords:** climate change, common garden, shrubs, functional traits, ecophysiology, phylogenetic niche conservatism

## Abstract

Leaf drought tolerance traits influence plant survival in water deficit conditions, and these traits are influenced by both the plant’s evolutionary history and the environment in which the plant is currently growing. However, due to the substantial phenotypic plasticity in leaf traits, we still do not know to what degree variation in leaf traits is governed by species’ phylogenetic history or by their environment. To explore this question, we re-examined a drought tolerance dataset from 37 native Australian shrub species with varying climate origins growing in a common garden located in Melbourne, Australia. We previously measured seven leaf morphophysiological traits, and here, we estimated how phylogenetically conserved these traits are. We quantified phylogeny and the strength of correlation between the morphological traits and physiological traits before and after accounting for shared phylogenetic history. We also evaluated the relationship between species’ leaf traits and the climate of their native ranges. We present three main findings: (a) most leaf drought tolerance traits had weak phylogenetic signals, which is consistent with the convergent evolution of these traits. (b) There is weak but consistent coordination between distinct leaf drought tolerance traits, which can be masked due to species’ phylogenetic histories. (c) Leaf drought tolerance traits show strong correlations with the climate of species’ origins, and this relationship is only weakly impacted by phylogenetic signals. Therefore, the role of phylogeny on the coordination among leaf functional traits and their links to climate were limited. A better understanding of trait–environment relationships might be more pivotal than understanding the evolution of these traits for improving the predictions of species’ response to climate change–type drought, especially for shrub species that span substantial aridity gradients.

## Introduction

Climate change is predicted to alter the water availability for plants worldwide (Sheffield and Wood, 2008; Dai, 2013). An increase in the frequency and severity of drought events poses serious challenges to natural vegetation, and improved theory and practices are needed for predicting species’ responses to such events (Bartlett et al., 2012). Functional traits reflect plants’ adaptations to their environment and have been widely applied to understand their key ecological strategies (Falster et al., 2018; Campetella et al., 2020; Krishna et al., 2021), including adaptations to drought conditions (Blackman et al., 2014; Bourne et al., 2017; Fletcher et al., 2018). For example, earlier studies have proposed that across global aridity gradients and biomes, plant species are structured along trait economics spectra for different above- and below-ground organs and that govern plant responses to environmental resources such as water, carbon, and nutrients (Reich, 2014; de la Riva et al., 2016; Vleminckx et al., 2021). At the resource-rich end of the spectrum, species generally show functional traits such as high specific leaf area (SLA) and high rates of resource uptake and exchange, which promote a faster return of investment but intolerance to low resources (Wright et al., 2004; Heilmeier, 2019 and their cited references). In contrast, resource-poor conditions tend to favor species with traits such as low SLA, high tissue density, and longer leaf lifespan, which confer a slow return of investment but higher tolerance to abiotic stress such as drought (Chen et al., 2021; Krishna et al., 2021). Moreover, the shifts of trait values at the community scale are not invariably coherent and may not accurately represent shifts in functional traits and the performance or fitness of all species (Derroire et al., 2018; Kandlikar et al., 2018). Additionally, the modulation of functional traits and trait relationships by climate is surprisingly modest (Wright et al., 2004; Heilmeier, 2019). There is still a pressing need for understanding plant function and physiology across aridity gradients as climate change is already impacting vegetation dynamics, composition, and distribution.

In addition to environmental factors, plant species‘ evolution history is an important factor that constrains species’ functional traits (Zhang et al., 2011). In general, variations in functional traits among closely related species tend to be small, while those with distant phylogenetic relationships can be much larger (Felsenstein, 1985), although there is also evidence that across larger phylogenetic distances, we can expect both large and small trait differences, depending on the nature of trait evolution (Tucker et al., 2018). Some species with close evolutionary relationships that evolved under different environmental conditions are more affected by the heterogeneous environment than by their evolutionary history (Blomberg et al., 2003). Contrarily, convergent evolution to shared environmental conditions may make distant species show similar functional traits (Wake, 1991). It is thus necessary to consider the phylogenetic relationship between species when studying the correlation of traits among species, that is, to test whether the functional traits of species show phylogenetic signals (Ackerly and Reich, 1999). Additionally, plant phylogenetic backgrounds also inevitably confound the effect of climate on trait variation (Li et al., 2016). Currently, the influence of phylogenetic relationships of plant species on the coordination among functional traits and their links to climate remain unclear.

Leaf morphological and physiological traits are closely related to the mechanisms of drought tolerance and therefore deemed as pivotal characteristics for determining species’ drought adaptive capacity (Delzon, 2015; Fletcher et al., 2018). For example, a smaller leaf facilitates cooling (Wright et al., 2017), lower SLA improves leaf economic return under hot and dry climates (Wright et al., 2004; Costa-Saura et al., 2016), a higher Huber value (HV) maintains the capacity of stems to transport water to leaves (Vander Willigen and Pammenter, 1998; Carter and White, 2009), more negative osmotic water potential at zero turgor (π_tlp_) facilitates the maintenance of turgor and stomatal aperture under worse soil moisture conditions (Maréchaux et al., 2015; Zhu et al., 2018), and more negative water potential values inducing 50% loss in leaf hydraulic function define the boundaries of plant distribution (Blackman et al., 2014; Nardini and Luglio, 2014). As the above literatures reported, differences in drought tolerance mechanisms relate to leaf drought tolerance traits; it is necessary to include these traits in drought tolerance analysis. Nevertheless, due to the perplexing ecological factors, diverse plant life forms and function types, plant traits’ plasticity, and phylogenetic non-independence of study species, which leaf trait or trait association better stands for drought tolerance is an unresolved issue (Perez and Feeley, 2020; Xu et al., 2020).

Previous studies have shown contradictory relations between the origin climates of species and their drought tolerance. While there is a long history of work linking species drought tolerance traits to their climatic origins (Blackman et al., 2014; Costa-Saura et al., 2016; Zhu et al., 2018), it is still unclear how species’ climate origins determine the contribution to the physiological and functional traits of plants growing in shared environmental conditions (e.g., Warren et al., 2005; Knutzen et al., 2015). Part of the challenge may be due to species’ shared phylogenetic histories, which can constrain both species’ mean trait values and the extent of trait plasticity (Munzbergova et al., 2017). In our previous work, we screened the 37 shrub species originating from different climatic environments across Australia but grown in a common field environment at the Burnley Campus of the University of Melbourne (**Supplementary Table 1**), to quantify the relationships between species’ climatic origins and their leaf morphophysiological traits (Xu et al., 2020). In that study, we found that, among seven leaf drought tolerance traits, leaf sizes (LSs), HVs, the osmotic potentials at full turgor (π_0_), turgor loss point (π_tlp_), and midday leaf water potential (*Ψ*_mid_) were significantly correlated with species’ climate origins. Here, we extend the previous work by evaluating the phylogenetic context to understand the evolution of leaf drought–tolerant traits, a critical step for predicting the effects of climate change on species and ecosystems (Fletcher et al., 2018). In particular, we reanalyzed the data published in Xu et al. (2020) and explored the influence of phylogeny on the relationship between leaf morphological and physiological traits and their link to the climate of origin, with the aim to determine whether phylogenetic signals affect relationships among leaf drought tolerance traits and between traits and climate envelope. We believe that the current study can further our understanding of the conflicting relationships between the climate of origin and plant drought tolerance traits.

## Materials and methods

### Site description

As detailed in Xu et al. (2020), we measured a suite of leaf drought tolerance traits for 37 shrub species growing under the same environmental conditions on the Burnley Campus of University of Melbourne, Australia (37◦47’ S; 144◦58’ E). The multiyear average maximum and minimum temperature (1856–2014) at the Burnely Campus is 19.87°C and 10.27°C, and the multiyear average rainfall (1856–2014) was 648.30 mm (the data are from Olympic Park, located 3 km away from the study site; Australian Bureau of Meteorology, http://www.bom.gov.au/climate/data/). The individuals of the 37 focal species, all of which were native to the Australian continent, were acquired from commercial nurseries and planted in the field at the study site. Among these shrubs, the vast majority of seedlings were transplanted between 2009 and 2011 to the Burnley Campus. The contemporary ranges of these plants encompass a wide range of environmental conditions, with a mean annual precipitation (MAP) ranging from approximately 300–1,200 mm [for detailed climatic ranges for each species, refer to Xu et al. (2020)]. The Burnley Campus thus serves as an experimental common garden, where plants native to a wide range of environmental conditions grow together in a shared environment. We measured all traits during the cool winter season (13–30 June 2016) to avoid any transient plasticity that might occur during the summer drought.

### Climate variables

With the aim to investigate the influences of the climate of provenance on leaf traits, we obtained the records of natural distribution occurrence and corresponding climatic parameters from the Atlas of Living Australia (website available at http://www.ala.org.au). The validated records of species occurrence layers were overlaid with the selected environmental layers and then downloaded from the ALA using the “mapping and analysis” portal. The climatic layers (based on a gridded dataset, ~1 km × 1 km) were extracted from the environmental layer portal on the ALA. For a detailed description, refer to the article published by Xu et al., 2020. We used mean annual precipitation (MAP) and mean annual aridity index (AI) for all occurrence points across each species distribution within Australia as the key climate variables for analysis. As the dry extremes are generally the key limits for plant survival under drought conditions across Australian vegetation types (Mitchell et al., 2014), we characterized the dry extremes of each species’ range as the 5th percentile values of the MAP and AI.

### Measurements of leaf drought tolerance traits

In this study, we reanalyzed data on seven leaf traits, previously published in Xu et al. (2020). Namely, we focused our analyses on three morphological traits related to leaf drought tolerance—LS, SLA, HVs (sapwood-to-leaf area ratio), and four physiological traits—osmotic potential at full turgor point (π_o_), the bulk leaf turgor loss point (π_tlp_), elastic modulus at full turgor (*ϵ*), and midday leaf water potential (MWP, *Ψ*_mid_).

For the morphological traits, we removed all the leaves of one small shoot from three or four replicate individuals per species and counted the leaf numbers per shoot. We scanned the leaf area of each shoot with an LI3100 area meter (Li-Cor, Lincoln, NE, USA). LS was calculated as the total leaf area on a shoot divided by the number of leaves on that shoots, and the mean LS per individuals was calculated as the average of the three or four shoots. We then dried leaves at 70°C until constant weight in a drying oven, after which we calculated SLA as fresh leaf area divided by dry mass. The small shoots were also used for calculating the HVs, measured as the total cross-sectional area-to-leaf area ratios (including all the leaves on the small shoots). For the small shoots, the heartwood areas were neglected.

We measured pressure–volume (P-V) curves with a minimum of four repeats per species using the bench-drying method (Tyree and Hammel, 1972). For P-V curve measurements, the shoots of six individual shrubs were cut in the morning (04:00–05:00) and rehydrated in deionized water for at least 1 h. When the leaf water potential was less than -0.1 MPa, we considered that the leaf could not fully rehydrate and then discarded it. The turgor traits were gained from P-V curves. For the midday leaf water potential (*Ψ*_mid_) measurements, leaves were excised and immediately sealed in ziplock bags and stored in a cooler for transport to the laboratory (Rodriguez-Dominguez et al., 2022). Measurements began within 10 minutes and finished within 30 minutes of leaves collection. A Scholander-type pressure chamber (Soil moisture Equipment Corp., Santa Barbara, CA, USA) was used to measure leaf water potential for P-V curves and *Ψ*_mid_.

### Phylogenetic and statistical analyses

We used the package *V.PhyloMaker* in R to build a phylogenetic tree of the focal species (n = 37) (Jin and Qian, 2019). This package produces phylogenies for vascular plants using two mega-trees as a backbone (Zanne et al., 2014; Smith and Brown, 2018). Based on the phylogeny, we plotted the taxon-specific features and climate conditions of natural distribution with annotated layers by the *ggtreeExtra* package (Xu et al., 2021; **Supplementary Figure 1**). We conducted a principal component analysis (PCA) using *FactoMineR* (Le et al., 2008) and *factoextra* packages (Kassambara and Mundt, 2020) to survey the covariation of multivariate traits among the shrubs after leaf functional traits were z-transformed (xi – x̅/standard deviation), where xi was the average measurement value for specific leaf trait of shrub i and x̅ was the average value of the 37 shrub species for the specific leaf trait i. Eigenvectors with values greater than 1 were selected as principal components. The ‘pic’ function in the *ape* package in R was used to carry out phylogenetically independent contrasts (PICs), which results in 36 (n-1) contrasts (Paradis and Schliep, 2019). A phylogenetic PCA was also conducted after correcting for phylogeny using PIC. The Pearson correlations of the PIC values for each trait were used to estimate phylogenetically corrected relationships among traits. Phylogenetic signals were identified and tested using the phylosignal package in R (Keck et al., 2016). The indexes of phylogenetic signal comprise Moran’s I index, Abouheif’s C_mean_ index, Blomberg’s K and K*, and Pagel’s λ. To graphically represent how the data are autocorrelated at different lags of distance, phylogenetic correlograms for leaf traits were carried out (Keck et al., 2016). To investigate the influences of the climate of native distribution on leaf drought tolerance traits before and after accounting for phylogeny, we used linear regression from the packages of *ggplot2*, *dplyr*, *ggpubr*, *ggmisc*, and *MASS*. We also corrected for the influences of phylogeny on climate parameters. We did so because climate data were obtained from the coordinates of the distribution of extant plant species and were the results of the long-term evolution of these plants (Zhang et al., 2011). We assessed the correlations among leaf drought tolerance traits and among their PIC across shrubs species by constructing a Pearson correlation matrix using the gparirs package. All data analyses and graphing were carried out by R ver. 3.6.3 (R Core Team, 2021).

## Results

### Phylogenetic basis and phylogenetic signal of leaf traits

Leaf functional traits showed a wide range of variation across 37 shrub species, even though shrubs were grown in a common environment (**Table 1**). LS showed the most variation (CV=144.60%), while osmotic potential at the turgor loss point (π_tlp_) showed the least variation (CV=21.92%).

**Table 1:** Summary of the seven leaf drought tolerance traits of the 37 shrub species included in this study.

Trait	Abbreviation	Unit	Range (min–max)	Coefficient of variation (%)
Leaf size	LS	cm^2^	0.03–17.92	144.60
Specific leaf area	SLA	cm^2^ g^-1^	12.22–308.89	53.06
Huber value	HV	cm^2^ m^-2^	2.62–18.38	42.33
Elastic modulus at full turgor	ϵ	MPa	2.55–11.27	34.58
Midday water potential	*Ψ*_mid_	MPa	-2.78–0.65	33.57
Full turgor point	π_0_	MPa	-2.25–0.9	22.25
Turgor loss point	π_tlp_	MPa	-2.85–1.27	21.92

**Figure 1:**
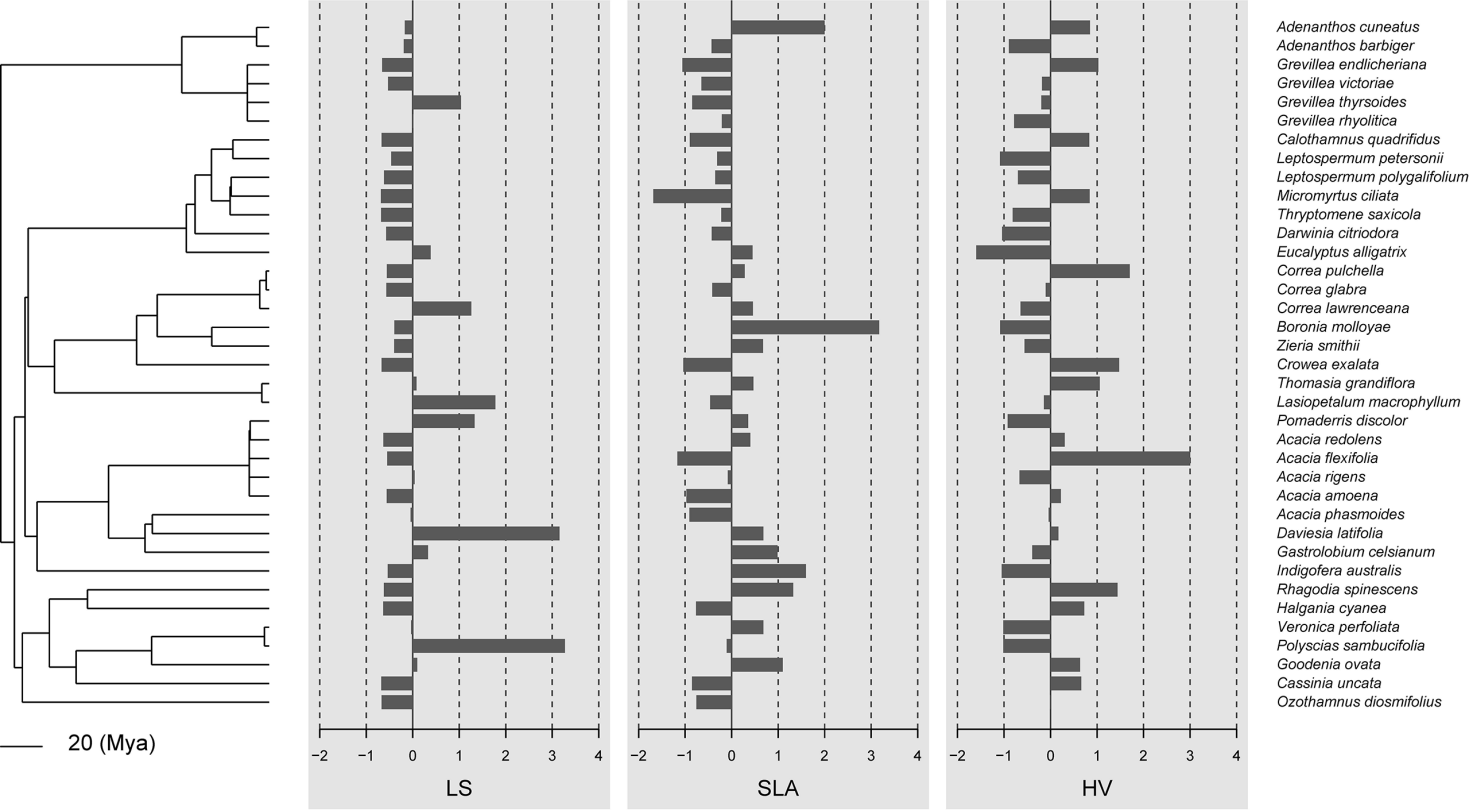
Data visualization of the three morphological traits (leaf size, specific leaf area, and Huber values) mapped along the phylogeny of 37 shrub species. If these traits were phylogenetically conserved, closely related species should share similar bar lengths. By default, data are centered and scaled by trait.

**Figure 2:**
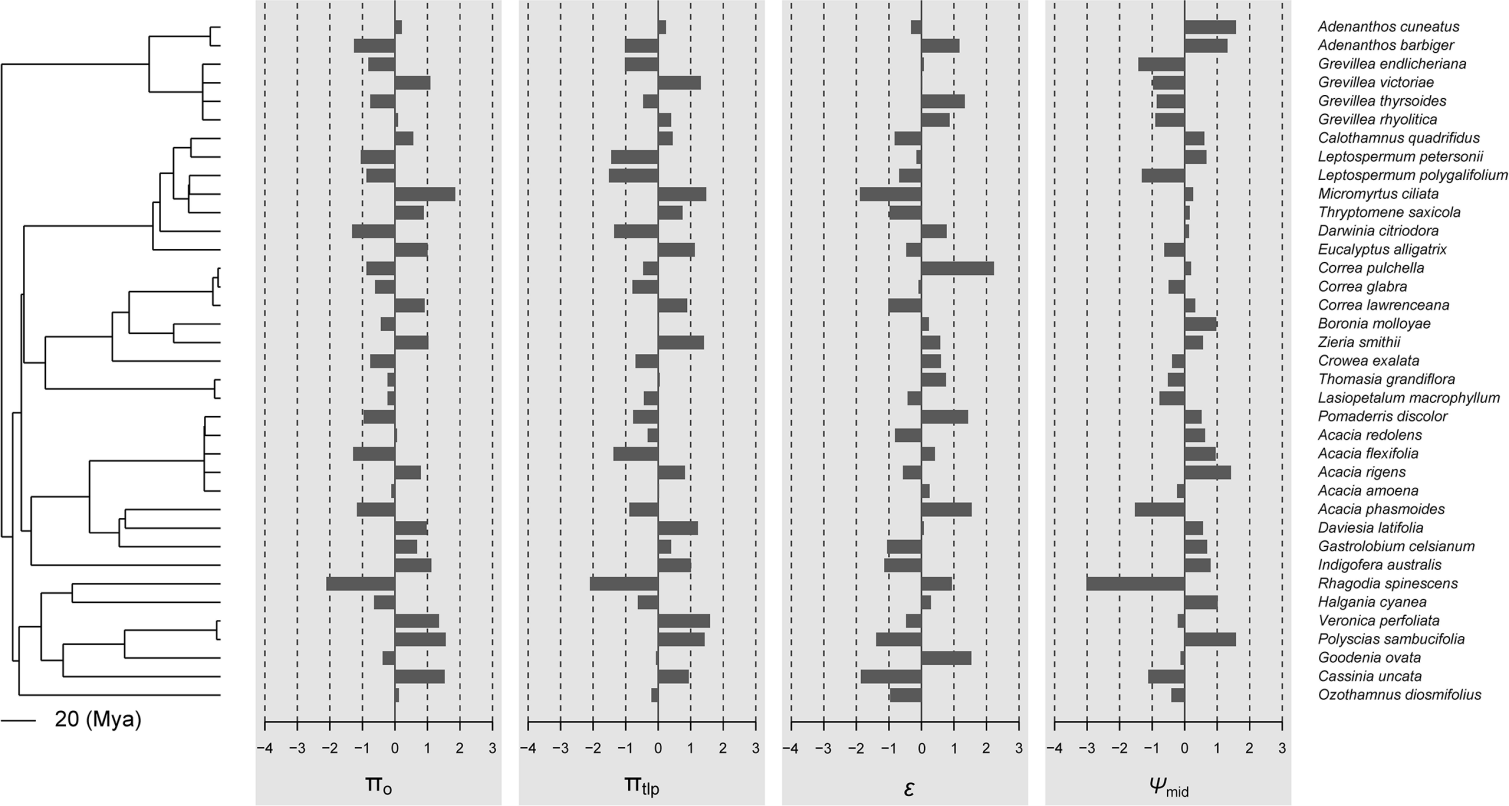
Data visualization of the four physiological traits (π_o_, π_tlp_, *ϵ*, and *Ψ*_mid_) mapped along the phylogeny of 37 shrub species. If these traits were phylogenetically conserved, closely related species should share similar bar lengths. By default, data are centered and scaled by trait.

**Figure 3:**
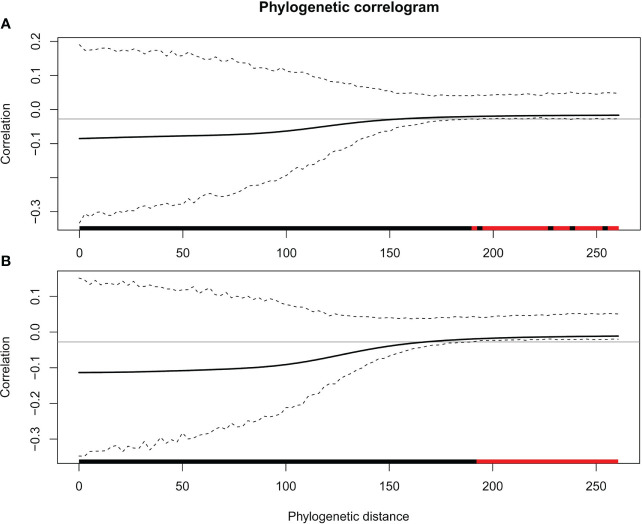
Phylogenetic correlograms for two traits: **(A)** π_o_ and **(B)** π_tlp_. The solid bold black line represents the Moran’s I index of autocorrelation, and the dashed black lines represent the lower and upper bounds of the confidence envelope (95%). The horizontal black line indicates the expected value of Moran’s I under the null hypothesis of no phylogenetic autocorrelation. The colored bar shows whether the autocorrelation is significant based on the confidence interval (red) or not (black).

In general, there was little evidence that leaf traits are phylogenetically conserved among the 37 species in our study (**Figures 1**, **2**; **Table 2**). No traits showed significant phylogenetic signals using the C_mean_, I, or λ indices (*P* ≥ 0.153, *P* ≥ 0.102, and *P* ≥ 0.542, respectively). A significant phylogenetic signal (*P* < 0.05) was only detected for midday leaf water potential (*Ψ*_mid_, with Blomberg’s K and K* values of 0.246 and 0.253, respectively). The K values of other leaf traits showed remarkable lability and are independent of phylogeny. The zero value of lambda (λ) indicates that SLA, HV, π_o_, and π_tlp_ have evolved independently of phylogeny (**Table 2**). The intermediate values of λ between 0 and 1 for LS, *ϵ*, and *Ψ*_mid_ (**Table 2**) suggest that, although influenced by phylogeny, these traits may have evolved through a process other than random drift. The phylogenetic correlogram of most leaf functional traits are flat and not significant (**Supplementary Figure 2**). The correlogram of π_o_ and π_tlp_ exhibits a strong positive autocorrelation for lags over 200 Mya (**Figure 3**).

**Table 2:** Computed phylogenetic signal indices and their respective *P*-values (in parentheses) for shrub drought tolerance traits.

Trait	Abouheif’s C_mean_	Moran’s I	Blomberg’s K	K*	Pagel’s λ
LS	-0.018 (0.370)	0.000 (0.233)	0.244 (0.105)	0.241 (0.120)	0.665 (0.542)
SLA	0.077 (0.153)	0.023 (0.193)	0.223 (0.054)	0.219 (0.065)	0.000 (1.000)
HV	-0.065 (0.631)	-0.060 (0.668)	0.095 (0.708)	0.098 (0.713)	0.000 (1.000)
π_o_	-0.106 (0.755)	-0.046 (0.564)	0.152 (0.259)	0.155 (0.223)	0.000 (1.000)
π_tlp_	-0.086 (0.709)	-0.062 (0.681)	0.164 (0.168)	0.168 (0.150)	0.000 (1.000)
*ϵ*	-0.054 (0.569)	0.007 (0.287)	0.083 (0.835)	0.084 (0.829)	0.043 (0.785)
*Ψ*_mid_	0.058 (0.199)	0.053 (0.102)	0.246 (**0.031**)	0.253 (**0.026**)	0.579 (1.000)

C_mean_ is a measure of autocorrelation of the covariation of trait values relative to the phylogenetic distances between the species. C_mean_ = 1 signifies strong resemblance across close relatives, and C_mean_ = − 1 signifies a robust negative correlation between species and trait resemblance. The values of Blomberg’s K suggest that trait discrepancy across a phylogeny is discernible from Brownian motion. K > 1 suggests that phylogeny forecasts more trait difference than expected given Brownian motion due to trait conservation. K < 1, phylogeny forecasts less trait difference than expected under Brownian motion owing to phylogenetic trait convergence (Perez and Feeley, 2020). Pagel’s λ stands for a quantitative assessment of the phylogenetic signal for a trait and value of λ between 0 and 1. λ = 0 suggests that the trait has evolved independently from phylogeny. λ = 1 suggests a robust phylogenetic signal. The intermediate values of λ indicate that trait may have evolved by a process different than random drift (Krishna et al., 2021).

Significant values (p < 0.05) were shown in bold.

### Phylogenetic principal component analysis

**Figure 4:**
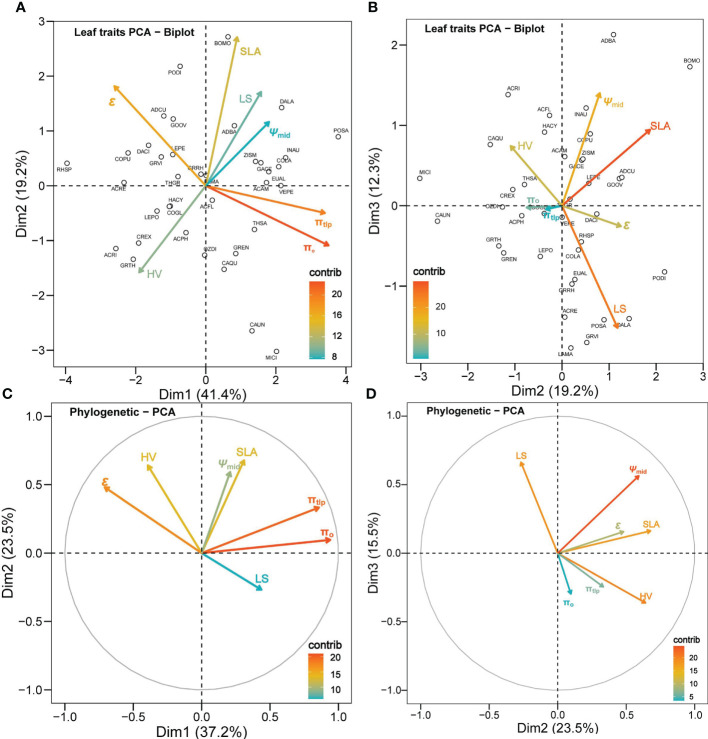
Principal component analysis (PCA) for the seven leaf traits of 37 shrub species. Panels **(A, B)** are not correcting for phylogeny, while **(C, D)** are a phylogenetically corrected PCA. The color gradients of the legend in each panel show the contribution of a trait to a given principal component in percentage. The hollow circles labeled with a four-letter species code aside in panels **(A, B)** represent each plant species (see supp. table 1 for full species names and corresponding species code).

Leaf drought tolerance traits showed substantial collinearity. The first axis of the non-phylogenetic PCA accounted for 41.4% of the variation, the second PCA axis accounted for 19.2% of the total variance, and the third PCA accounted for 12.3% of the total variance (**Figures 4A, B**). The first PC axis was positively correlated to π_o_ and π_tlp_ (r = 0.93 and 0.91, respectively), and negatively correlated to *ϵ* (r = -0.70). The second PC axis was positively correlated to SLA and *ϵ* (r = 0.73 and 0.48, respectively) and negatively correlated to HV (r = -0.42). In the phylogenetically corrected PCA, the first three axes accounted for 37.2%, 23.5%, and 15.5%, correspondingly, of the total variance in the phylogenetic correcting traits among the species (**Figures 4C, D**). The first PC axis was highly positively correlated to π_o_ (r = 0.94) and π_tlp_ (r = 0.86) and negatively correlated to *ϵ* (r = -0.71). The second PC axis was positively correlated to SLA (r = 0.68) and HV (r = 0.64) and negatively correlated to LS (r = -0.27). The third PC axis was positively correlated to LS (r = 0.66).

### Phylogenetic corrected trait–environment relationships

**Figure 5:**
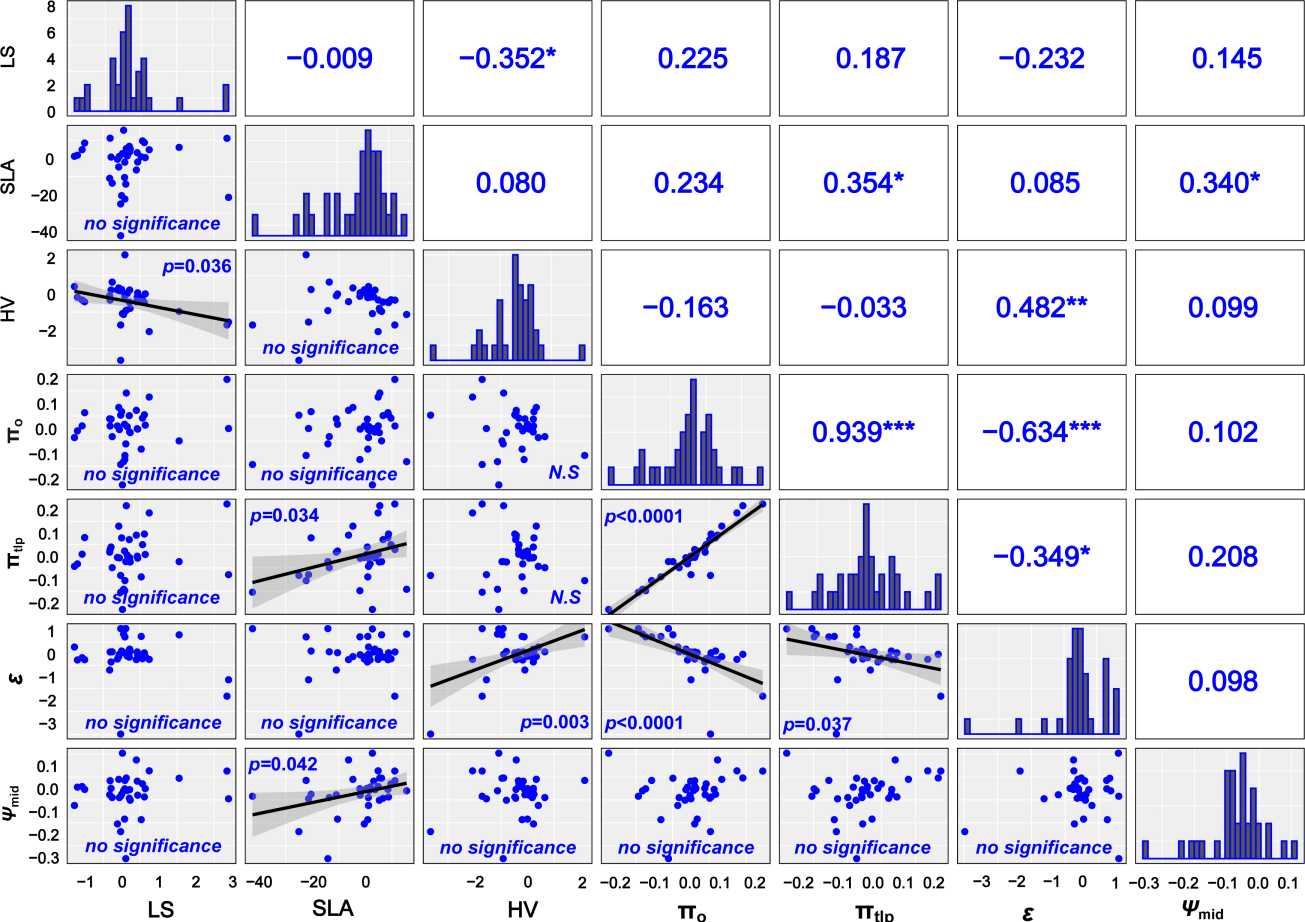
Correlation between leaf drought–tolerant traits after removing the phylogenetic signals. Solid lines represent the linear regressions, and shallow gray bands represent 95% confidence in the lower triangular intervals. The correlation coefficients are given respectively in the graphics above the diagonal. Histograms showing trait value distributions are given in the diagonal. *P < 0.05, ** *P* < 0.01, *** *P* < 0.001. N.S., non-significant relationship.

Without considering phylogenetic effects, there were no correlations between most functional traits (**Supplementary Figure 3**), except the physiological traits π_o_, π_tlp_, and *ϵ*. After accounting for shared phylogenetic history, we found that LS and HV were negatively correlated and SLA was positively correlated with π_tlp_ and *Ψ*_mid_ (**Figure 5**).

**Figure 6:**
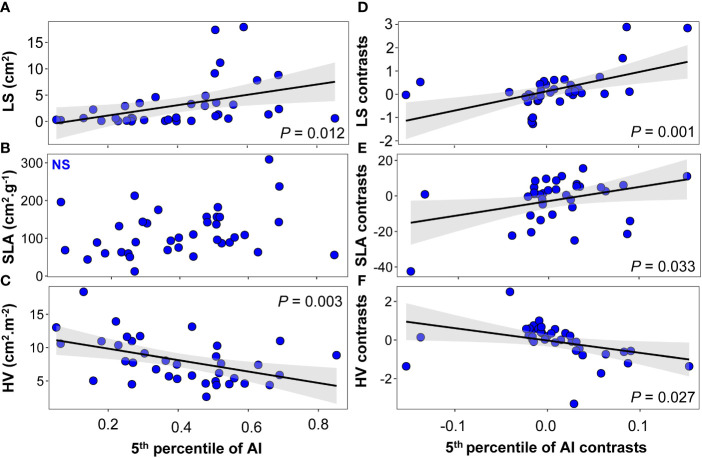
Relationships between morphological traits and the 5th percentile of the aridity index (AI) at the species level: **(A–C)** linear regression between morphological traits and the 5th percentile of AI; **(D–F)** phylogenetically independent contrast (PIC) linear regression between morphological traits and the 5th percentile of AI. Solid black lines indicate the linear trends of the 5th percentile of the AI changes of morphological traits, and shallow gray bands represent 95% confidence intervals. N.S., non-significant relationship.

**Figure 7:**
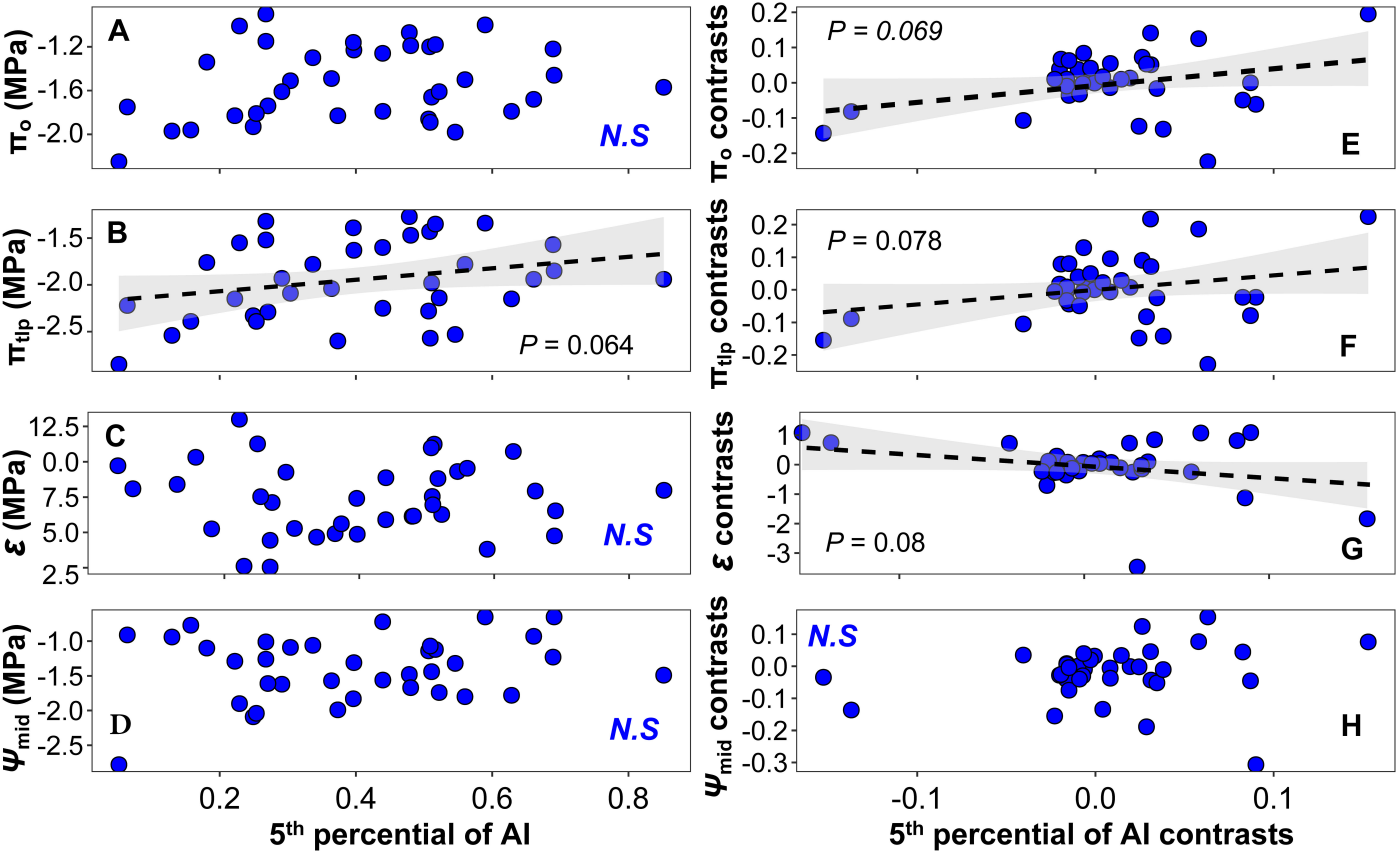
Relationships between physiological traits and the 5th percentile of AI at the species level: **(A–D)** linear regression between physiological traits and the 5th percentile of AI; **(E–H)** PIC linear regression between physiological traits and the 5th percentile of AI. Black dashed lines indicate marginally significant linear trends of the 5th percentile of the AI changes of physiological traits, and shallow gray bands represent 95% confidence intervals. N.S., non-significant relationship.

We observed a significant linear relationship between LS, HV, and mean climate variables including MAP (LS: *F*=6.19, *P* =0.018; HV: *F*=17.90, *P* < 0.001; **Supplementary Figures 4A, C**), the 5th percentile of MAP (LS: *F*=8.24, *P* =0.007; HV: *F*=18.40, *P* < 0.001; **Supplementary Figures 6A, C**), and AI (LS: *F*=6.99, *P* = 0.012; HV: *F*=9.86, *P* =0.003; **Figures 6A, C**). As in Xu et al. (2020), we did not find a significant linear relationship between most physiological traits and the climate variables (**Figure 7** and **Supplementary Figures 5**, **7**). The exceptions to this were π_o_ and π_tlp_, which were marginally associated with MAP (π_0_: *F*=3.08, *P* =0.088; π_tlp_: *F*=3.26, *P* =0.08; **Supplementary Figures 5A, B**) and the 5th percentile of AI (π_tlp_: *F*=3.67, *P* =0.064; **Figure 7B**). When we controlled for phylogeny, we also found marginally significant correlations between four traits (SLA, π_0_, π_tlp_, and ϵ) and the 5th percentile of AI (SLA: *F*=4.93, *P* =0.033, **Figure 6E**; π0: F=3.53, P =0.069, **Figure 7E**; π_tlp_: *F*=3.31, *P* =0.078, **Figure 7F**; and ϵ: *F*=3.24, *P* =0.08, **Figure 7G**). We also found that *Ψ*_mid_ was marginally associated with the 5th percentile of MAP (*F*=43.45, *P* =0.072; **Supplementary Figure 7H**).

## Discussion

The drivers of interspecific variation in drought tolerance are vital for predicting and managing possible alterations in the ecosystem structure and functions under various scenarios of global change (Blackman et al., 2014; Delzon, 2015). Across large geographic scales, plants that are more exposed to drought often have a suite of resource-conservative traits, including smaller leaves (Wright et al., 2017), a low SLA (Mitchell et al., 2008; Nardini et al., 2012), higher HV (Macinnis-Ng et al., 2004; Martínez-Vilalta et al., 2009), shorter heights (Liu et al., 2019), and lower leaf area index (LAI, Asner et al., 2003). Plants in arid environments also tend to express more drought-tolerant physiological traits including lower turgor loss point [π_tlp_, (Mitchell et al., 2008; Bartlett et al., 2012; Nardini et al., 2012; Bartlett et al., 2014)], lower hydraulic vulnerability (Meinzer et al., 2009, Nardini et al., 2012), and stomata that close later during drought (Martin-StPaul et al., 2017; Zhu et al., 2018). However, we still know little about how evolutionary relatedness between species in arid environments affects observed correlations among traits and their links to climate variables.

Our study of how phylogenetic history shapes the drought tolerance of plants showed three main findings. (a) Most of the measured traits showed no phylogenetic signal, indicating that drought tolerance may be shaped more by species’ abiotic environment than evolutionary histories. When we found significant phylogenetic signals for traits (e.g., π_o_ and π_tlp_), this signal was weak, again indicating substantial lability. (b) Surprisingly, we found only a moderate-to-weak correlation between leaf drought tolerance traits, even after taking species’ phylogeny into account. This suggests that while all measured traits can contribute to species’ overall drought tolerance, there may be independent evolution between traits, which can allow plants to adopt a wider set of trait combinations. (c) Phylogenetic relatedness has no apparent influence on the relationship between leaf drought tolerance traits and climate variables, which again indicates that species can evolve drought tolerance independently of their main lineages.

### Evolution and correlation of leaf drought tolerance traits

Our study indicated that most tested leaf drought tolerance traits have a weak phylogenetic signal. These results suggest that leaf drought tolerance traits are not phylogenetically conserved and are more consistent with the hypothesis of convergent evolution (**Figures 1**, **2**). The present study indicates that phylogenetic independent contrasts (PICs) explain little of the variation in the leaf drought tolerance traits of shrub species occurring along aridity gradients.

The tight positive relationship between osmotic potential at π_o_ and π_tlp_ and their link to *ϵ* were general and supported in other recent analyses (Bartlett et al., 2012; Nardini and Luglio, 2014). There were a few correlations that only emerged after using the PIC. LS, SLA, HV, π_tlp_, and *ϵ* were significantly correlated after correcting for phylogeny (**Figures 5**, **6**). Therefore, the phylogenetic signal in LS, SLA, HV, π_tlp_, and *ϵ* does influence the correlation between drought tolerance traits and masks the coordination relationship between some functional traits. Although a growing body of literature indicates that smaller SLA and lower π_tlp_ have developed rather separately under diverse selection pressures (Zhu et al., 2018; Majekova et al., 2021 and references therein), our current results do not support this hypothesis as there was a significant positive correlation between SLA and π_tlp_ after removing the phylogenetic effect (**Figure 5**).

### Evolutionary association of traits with climate

A growing body of literature documents coordination in plant functional traits and patterns of shifts in trait values along single gradients (Read et al., 2014; Wright et al., 2017). We found that many climate variables of species natural distribution were significantly correlated with leaf morphological traits after removing phylogenetic signals. We found support for a correlation of LS, SLA, and HV with the 5th percentile of AI in phylogenetic linear regression across set of 37 species (**Figures 6D–F**). Lower LS in drier environments is often observed (Fonseca et al., 2000), as LZ represents the evaporative surface and is a major driver of water losses in plants (Poorter et al., 2009). Even after taking into account the phylogenetical relatedness, our results showed a strong relationship between SLA and the 5th percentile of AI (**Figure 6**). Although previous research has demonstrated that there are no or only faint correlations between SLA and aridity gradients at various spatial scales and across taxonomic units (Bartlett et al., 2012), several studies have suggested that SLA should be seriously considered as a surrogate to plants’ drought strategy when forecasting the species future distribution and estimating their fitness to drier environments (Majekova et al., 2021). Because a leaf is a multifunctional organ, and SLA is an important leaf economics spectrum trait, this trait may not only reflect drought response but may also be influenced by other environmental factors such as light or nutrient availability. For example, at local scales, depending on the prevailing conditions, SLA can actually show the inverse relationship with water availability, where it may relate to the diversion of resources to promote root foraging (Wellstein et al., 2017; Ferguson et al., 2021). Our research indicated that drought tolerance traits π_o_, π_tlp_, and *ϵ* are adaptive in drier sites, as evidenced by phylogenetic least squares regressions with the 5th percentile of AI (**Figure 7**). Finally, in our current study, little attention was given to any potential genetic correlation structure present below the species level. If there is considerable local adaptation or another population structure within species, it is possible that ignoring such intraspecific genetic variation has obscured important phylogenetic trait patterns (e.g., McKay et al., 2003; Bolnick et al., 2011). Exploring such intraspecific trait and genetic variation should be a focus for future studies, especially in species with large geographic ranges that span substantial aridity gradients (Joly et al., 2019; Welles and Funk, 2021).

## Conclusions

Our research explored the diversification of leaf drought tolerance traits of shrubs in the Australian mainland. Most traits exhibited significant evolutionary lability and, to a large extent, were independent of phylogenetic history. Only a few correlations were apparent after taking phylogenetic information into account. Our results suggest that phylogeny has a limited ability to promote our understanding of the variation in shrub species’ leaf drought–tolerant traits across this aridity gradient in Australia. Our results underscore the importance of LS, SLA, HV, π_o_, π_tlp_, and *ϵ* as traits driving drought tolerance and suggest that a better understanding of contemporary trait–environment relationships might be more pivotal than understanding the evolution of these traits for improving the predictions of species’ response to ongoing and future drought.

## Data availability statement

The original contributions presented in the study are included in the article/**Supplementary Material**. Further inquiries can be directed to the corresponding authors.

## Author contributions

G-QX devised the idea and conducted the study work. G-QX drafted the manuscript. GK and MV provided conceptual advice. G-QX, GK, and MVanalyzed the data. All authors read and endorsed the final manuscript.

## Funding

The research work was financially assisted by Xinjiang Uygur Autonomous Region Tianshan Youth Program Project (No. 2020Q025), the National Natural Sciences Foundation of China (No. 32171874 and 41730638), and the Key Research Project of Frontier Sciences, CAS (No. QYZDJ-SSW-DQC014).

## Acknowledgments

We appreciate all the staffs at the Department of Ecosystem and Forest Sciences, University of Melbourne, for their excellent assistance. Special thanks to Prof. Stefan K. Arndt and Dr. Claire Farrell for their help with the lab work. We also thank Sanders Gregor for his assistance in collecting climatic data. Thanks to Christopher Szota, Sanders Gregor, and Lisa Wittick for preparing the pressure chamber and the pressure air.

## Conflict of interest

The authors declare that the research was conducted in the absence of any commercial or financial relationships that could be construed as a potential conflict of interest.

## Publisher’s note

All claims expressed in this article are solely those of the authors and do not necessarily represent those of their affiliated organizations, or those of the publisher, the editors and the reviewers. Any product that may be evaluated in this article, or claim that may be made by its manufacturer, is not guaranteed or endorsed by the publisher.
