# Supplementary material for: Evolutionary lability underlies drought adaptation of Australian shrubs along aridity gradients
Source: Front Plant Sci. 2022 Oct 7;13:949531. doi: 10.3389/fpls.2022.949531 (PMC9585297; doi:10.3389/fpls.2022.949531)
Supplement: Supplementary file 1 [file DataSheet_1.docx]

Supplementary Material

# Supplementary Tables

**Supplementary** **Table 1** Climate of origin for each shrub species grown in the common garden; mean annual aridity index (AI), mean annual precipitation (MAP) with the range, and mean annual temperature (MAT). Rows are ordered by increasing AI at the climate of origin.

| Species | Species code | AI | MAP (mm) | MAT (°C) | Median AI | Evergreen or deciduous | |
| --- | --- | --- | --- | --- | --- | --- | --- |
| *Grevillea victoriae* | GRVI | 1.381 | 1169 (631, 2004) | 10.77 | 1.189 | evergreen |  |
| *Correa lawrenceana* | COLA | 1.345 | 1172 (546, 2683) | 11.38 | 1.296 | evergreen |  |
| *Acacia phasmoides* | ACPH | 1.337 | 973 (614, 1200) | 12.69 | 1.254 | deciduous or evergreen |  |
| *Grevillea rhyolitica* | GRRH | 1.101 | 938 (864, 1016) | 14.98 | 1.127 | evergreen |  |
| *Polyscias sambucifolia* | POSA | 1.019 | 1120 (444, 2604) | 13.77 | 0.902 | evergreen |  |
| *Daviesia latifolia* | DALA | 1.017 | 954 (379, 2524) | 12.22 | 0.893 | evergreen |  |
| *Boronia molloyae* | BOMO | 1.006 | 923 (308, 1245) | 15.89 | 1.002 | evergreen |  |
| *Adenanthos barbiger* | ADBA | 0.984 | 891 (256, 1152) | 16.90 | 0.990 | evergreen |  |
| *Veronica perfoliata* | VEPE | 0.959 | 924 (409, 2368) | 11.19 | 0.806 | evergreen |  |
| *Goodenia ovata* | GOOV | 0.905 | 944 (196, 2524) | 13.64 | 0.876 | evergreen |  |
| *Leptospermum petersonii* | LEPE | 0.899 | 1334 (614, 2360) | 17.24 | 0.814 | evergreen |  |
| *Indigofera australis* | INDI | 0.867 | 844 (158, 2359) | 14.69 | 0.887 | evergreen |  |
| *Lasiopetalum acrophyllum* | LAMA | 0.826 | 895 (317, 1531) | 14.35 | 0.826 | evergreen |  |
| *Zieria smithii* | ZISM | 0.817 | 1181 (278, 3742) | 16.89 | 0.788 | evergreen |  |
| *Crowea exalata* | CREX | 0.757 | 884 (408, 2073) | 14.50 | 0.669 | evergreen |  |
| *Thomasia grandiflora* | THGR | 0.744 | 699 (345, 1099) | 17.35 | 0.781 | evergreen |  |
| *Eucalyptus alligatrix* | EUAL | 0.743 | 902 (459, 1312) | 13.57 | 0.714 | evergreen |  |
| *Leptospermum polygalifolium* | LEPO | 0.737 | 1113 (199, 3404) | 16.64 | 0.744 | deciduous |  |
| *Micromyrtus ciliata* | MICI | 0.711 | 685 (280, 2004) | 15.31 | 0.718 | evergreen |  |
| *Ozothamnus diosmifolius* | OZDI | 0.709 | 1024 (247, 2360) | 16.63 | 0.702 | evergreen |  |
| *Thryptomene saxicola* | THSA | 0.706 | 712 (362, 1181) | 15.93 | 0.683 | evergreen |  |
| *Grevillea endlicheriana* | GREN | 0.687 | 763 (350, 1087) | 18.09 | 0.666 | evergreen |  |
| *Pomaderris discolor* | PODI | 0.680 | 1002 (392, 1609) | 16.26 | 0.664 | deciduous |  |
| *Cassinia uncata* | CAUN | 0.613 | 779 (222, 2287) | 14.92 | 0.496 | evergreen |  |
| *Darwinia citriodora* | DACI | 0.579 | 836 (335, 1124) | 17.01 | 0.535 | evergreen |  |
| *Acacia amoena* | ACAM | 0.574 | 728 (524, 1380) | 13.68 | 0.485 | evergreen |  |
| *Adenanthos cuneatus* | ADCU | 0.560 | 643 (282, 1219) | 16.13 | 0.529 | evergreen |  |
| *Gastrolobium celsianum* | GACE | 0.487 | 506 (312, 1039) | 16.08 | 0.459 | evergreen |  |
| *Correa glabra* | COGL | 0.453 | 547 (229, 1233) | 15.28 | 0.424 | evergreen |  |
| *Acacia flexifolia* | ACFL | 0.424 | 577 (255, 1155) | 16.27 | 0.396 | Evergreen or deciduous |  |
| *Correa pulchella* | COPU | 0.422 | 461 (275, 835) | 16.23 | 0.334 | evergreen |  |
| *Calothamnus quadrifidus* | CAQU | 0.402 | 501 (195, 1252) | 17.84 | 0.384 | evergreen |  |
| *Grevillea thyrsoides* | GRTH | 0.371 | 476 (353, 653) | 18.71 | 0.398 | evergreen |  |
| *Acacia redolens* | ACRE | 0.312 | 397 (329, 675) | 16.24 | 0.298 | evergreen |  |
| *Acacia rigens* | ACRI | 0.226 | 323 (175, 818) | 16.94 | 0.214 | evergreen |  |
| *Halgania cyanea* | HACY | 0.214 | 329 (151, 1252) | 17.77 | 0.202 | evergreen |  |
| *Rhagodia spinescens* | RHSP | 0.175 | 313 (142, 1189) | 18.56 | 0.176 | evergreen |  |

Note: Median AI was calculated for each species distribution based on all available botanical/location records; the data was reproduced from previously published paper by Xu et al., 2020.

# Supplementary Figures


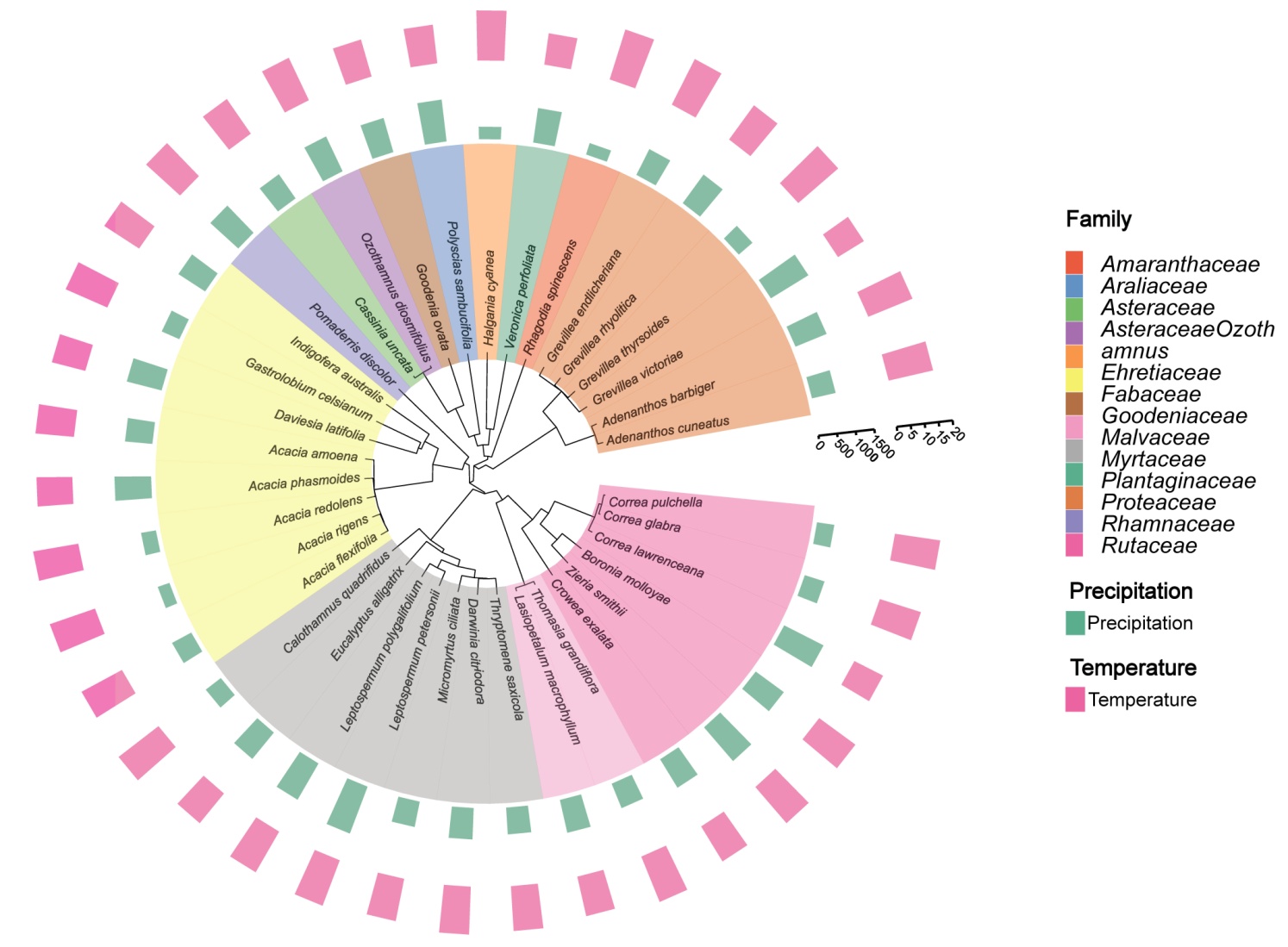


**Supplementary Figure 1.** Phylogenetic tree generated by V.PhyloMaker of the 37 shrub species.

**
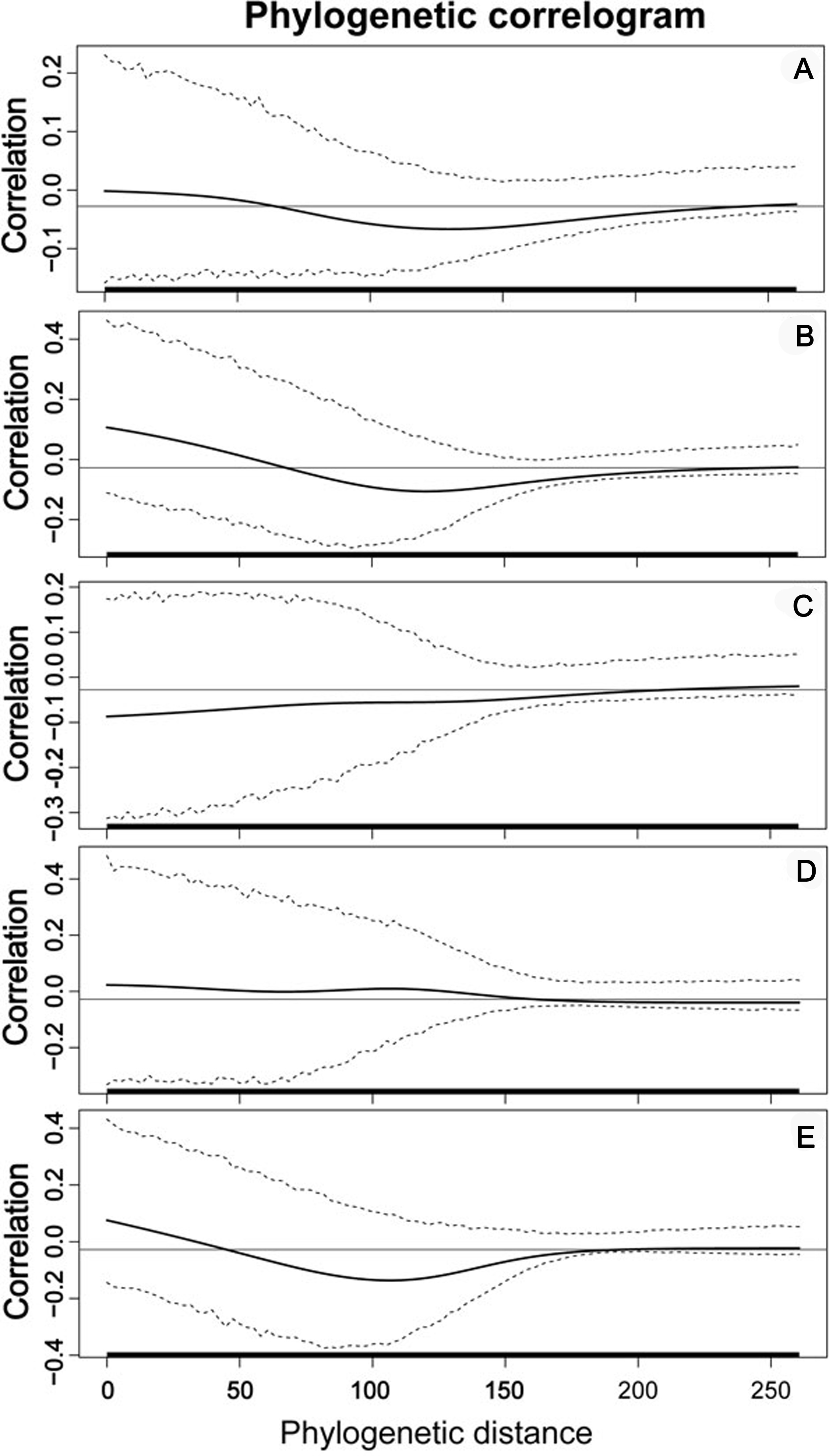
**

**Supplementary Figure 2.** Phylogenetic correlograms for 2 traits: (A) LS, (B) SLA, (C)HV, (D) *ε*, (E) *Ψ*_mid_. The solid bold black line represents the Moran’s I index of autocorrelation, and the dashed black lines represent the lower and upper bounds of the confidence envelope (95%). The horizontal black line indicates the expected value of Moran’s I under the null hypothesis of no phylogenetic autocorrelation. The colored bar show whether the autocorrelation is significant based on the confidence interval (red) or not (black).

**
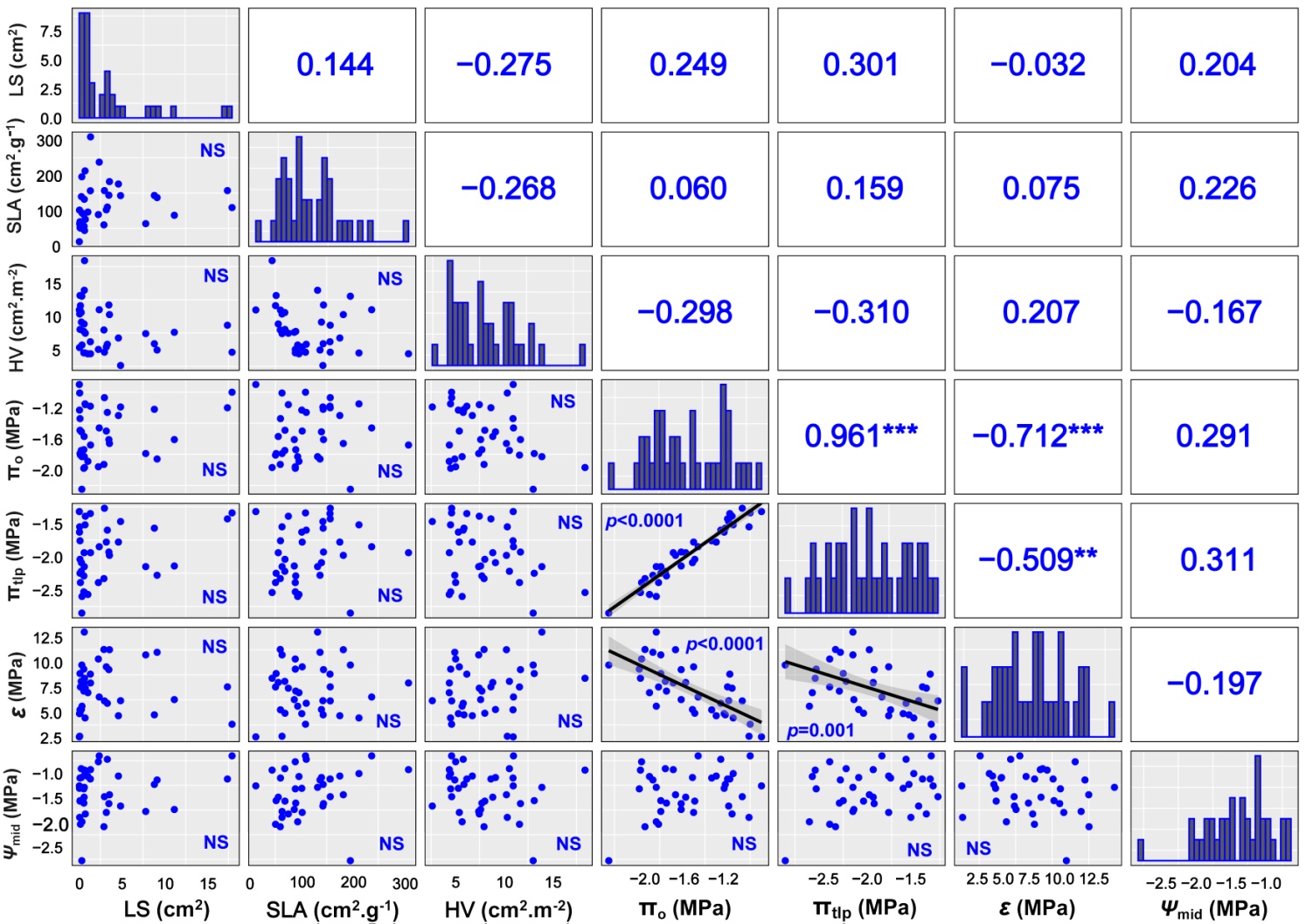
**

**Supplementary Figure 3.** Correlation between leaf drought tolerant traits without accounting for phylogenetic signals. Solid lines represent the linear regressions and shallow gray bands represent 95% confidence in the lower triangular intervals. The correlation coefficients are given respectively in the graphics above the diagonal. Histograms showing trait value distributions are given in the diagonal. **P* < 0.05, ** *P* < 0.01, *** *P* < 0.001. NS is for not significant.


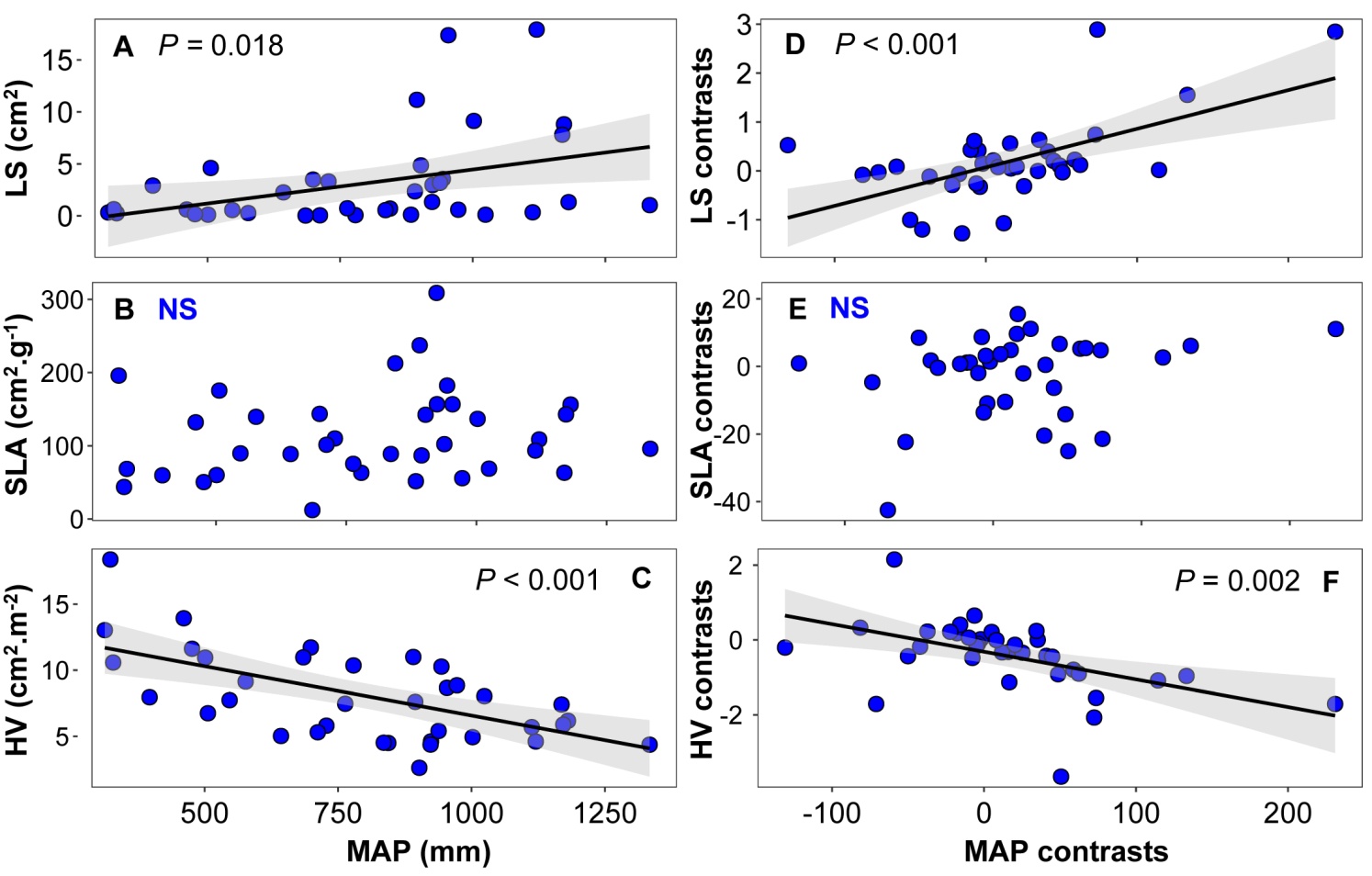


**Supplementary Figure 4.** Relationships between morphological traits and mean annual precipitation (MAP) at the species level: (A)-(C) Linear regression between morphological traits and MAP; (D)-(F) phylogenetically independent contrast linear regression between morphological traits and MAP. Black lines indicate significantly linear trends of MAP changes of morphological traits, and shallow gray bands represent 95% confidence intervals. NS is for not significant.


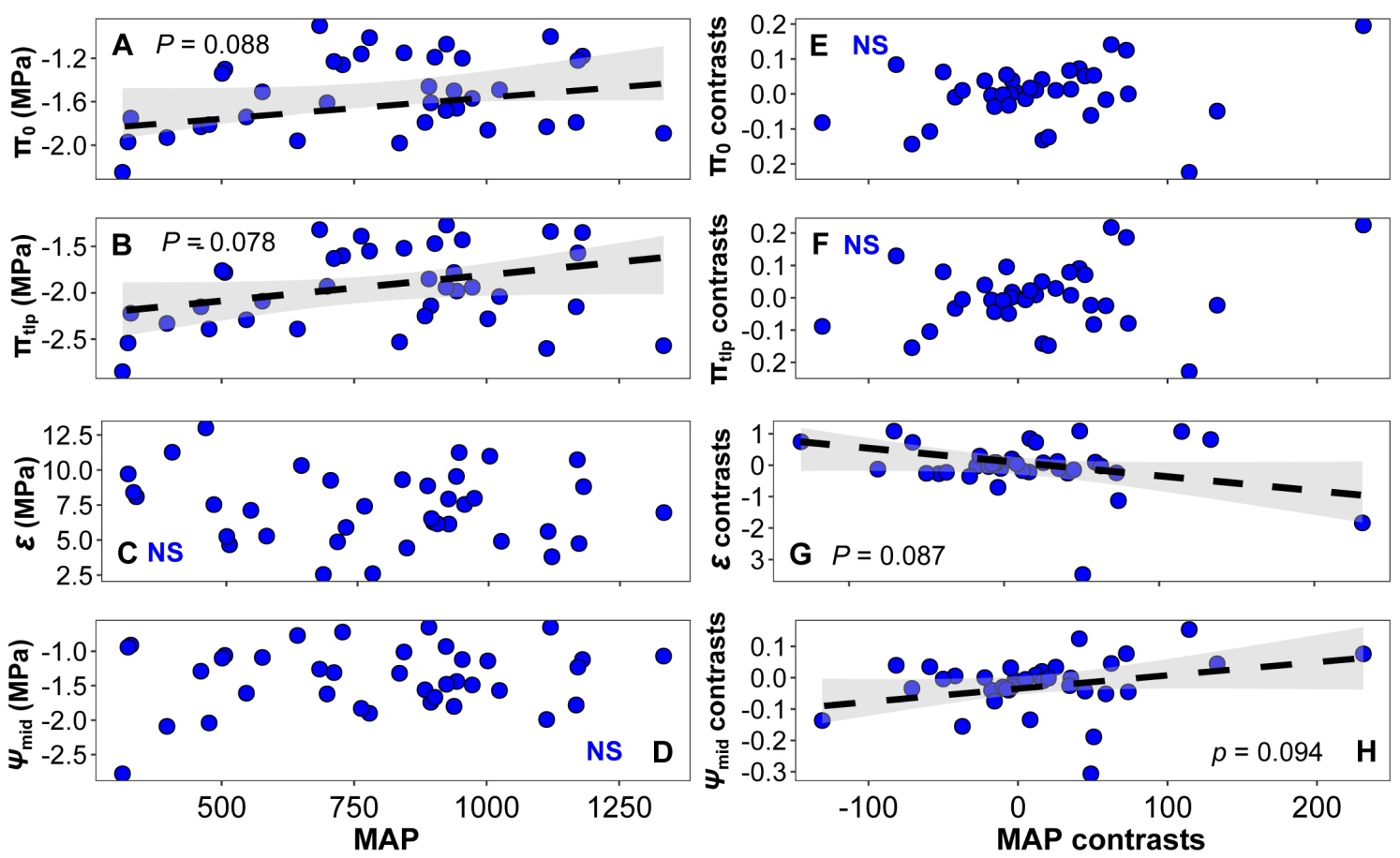


**Supplementary Figure 5.** Relationships between physiological traits and mean annual precipitation (MAP) at the species level: (A)-(D) Linear regression between physiological traits and MAP; (E)-(H) phylogenetically independent contrast linear regression between physiological traits and MAP. Black dashed lines indicate marginally significant linear trends of MAP changes of physiological traits, and shallow gray bands represent 95% confidence intervals. NS is for not significant.


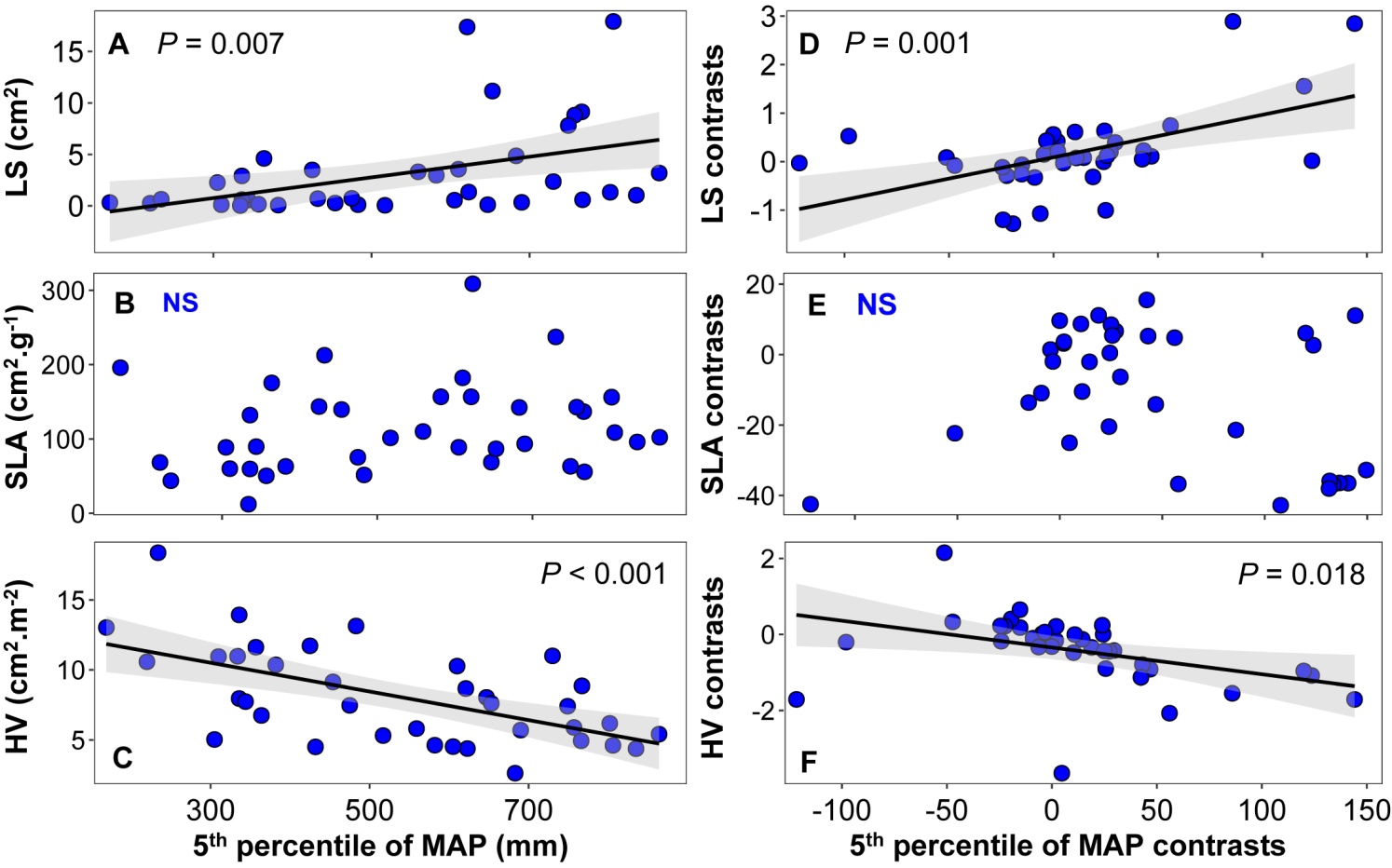


**Supplementary Figure 6.** Relationships between morphological traits and 5^th^ percentile of mean annual precipitation (MAP) at the species level: (A)-(C) Linear regression between morphological traits and 5^th^ percentile of MAP; (D)-(F) phylogenetically independent contrast linear regression between morphological traits and 5^th^ percentile of MAP. Black lines indicate significant linear trends of 5^th^ percentile of MAP changes of morphological traits, and shallow gray bands represent 95% confidence intervals. NS is for not significant.


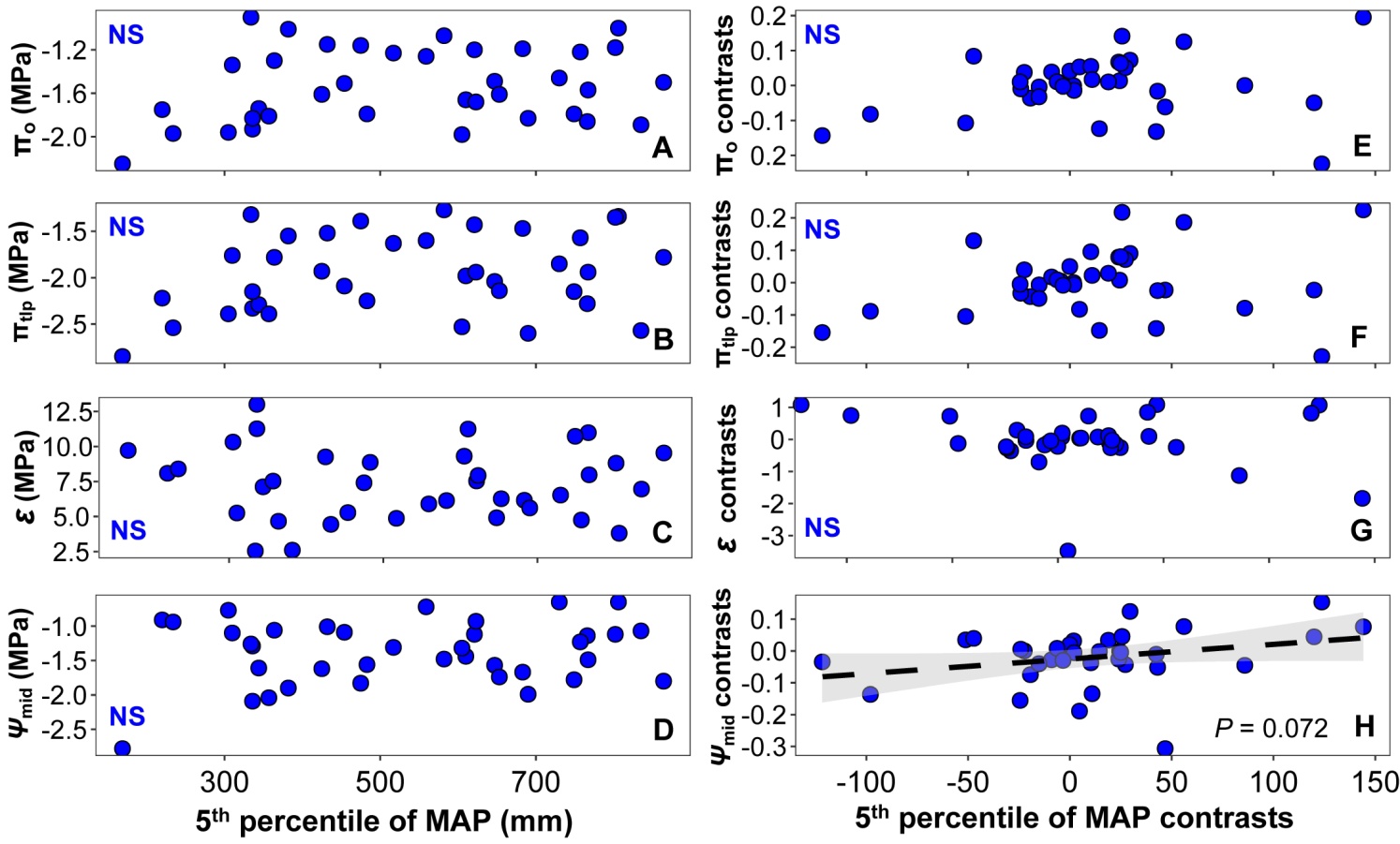


**Supplementary Figure 7.** Relationships between physiological traits and 5^th^ of mean annual precipitation (MAP) at the species level: (A)-(D) Linear regression between physiological traits and 5^th^ of MAP; (E)-(H) phylogenetically independent contrast linear regression between physiological traits and 5^th^ of MAP. Black dashed lines indicate marginally significant linear trends of 5^th^ of MAP changes of *Ψ*_mid_, and shallow gray bands represent 95% confidence intervals. NS is for not significant.
